# Single-nucleus RNA sequencing identifies cell-type–specific effects of sodium-glucose co-transporter 2 inhibitors in human myocardial slices

**DOI:** 10.1093/eurheartj/ehae472

**Published:** 2024-07-31

**Authors:** Kevin Schmidt, Maximilian Fuchs, Natalie Weber, Christopher Werlein, Jan D Schmitto, Fabio Ius, Arjang Ruhparwar, Christian Bär, Jan Fiedler, Thomas Thum

**Affiliations:** Institute of Molecular and Translational Therapeutic Strategies (IMTTS), Hannover Medical School, Carl-Neuberg-Str. 1, 30625 Hannover, Germany; Fraunhofer Institute for Toxicology and Experimental Medicine ITEM, Hannover, Germany; Fraunhofer Cluster of Excellence Immune-Mediated Diseases (CIMD), Hannover, Germany; Fraunhofer Institute for Toxicology and Experimental Medicine ITEM, Hannover, Germany; Fraunhofer Cluster of Excellence Immune-Mediated Diseases (CIMD), Hannover, Germany; Institute of Molecular and Translational Therapeutic Strategies (IMTTS), Hannover Medical School, Carl-Neuberg-Str. 1, 30625 Hannover, Germany; Center for Translational Regenerative Medicine, Hannover Medical School, Carl-Neuberg-Str. 1, 30625 Hannover, Germany; Institute of Pathology, Hannover Medical School, Hannover, Germany; Center for Translational Regenerative Medicine, Hannover Medical School, Carl-Neuberg-Str. 1, 30625 Hannover, Germany; Department of Cardiothoracic, Transplantation and Vascular Surgery, Hannover Medical School, Hannover, Germany; Center for Translational Regenerative Medicine, Hannover Medical School, Carl-Neuberg-Str. 1, 30625 Hannover, Germany; Department of Cardiothoracic, Transplantation and Vascular Surgery, Hannover Medical School, Hannover, Germany; Center for Translational Regenerative Medicine, Hannover Medical School, Carl-Neuberg-Str. 1, 30625 Hannover, Germany; Department of Cardiothoracic, Transplantation and Vascular Surgery, Hannover Medical School, Hannover, Germany; Institute of Molecular and Translational Therapeutic Strategies (IMTTS), Hannover Medical School, Carl-Neuberg-Str. 1, 30625 Hannover, Germany; Fraunhofer Institute for Toxicology and Experimental Medicine ITEM, Hannover, Germany; Fraunhofer Cluster of Excellence Immune-Mediated Diseases (CIMD), Hannover, Germany; Center for Translational Regenerative Medicine, Hannover Medical School, Carl-Neuberg-Str. 1, 30625 Hannover, Germany; Institute of Molecular and Translational Therapeutic Strategies (IMTTS), Hannover Medical School, Carl-Neuberg-Str. 1, 30625 Hannover, Germany; Fraunhofer Institute for Toxicology and Experimental Medicine ITEM, Hannover, Germany; Fraunhofer Cluster of Excellence Immune-Mediated Diseases (CIMD), Hannover, Germany; Institute of Molecular and Translational Therapeutic Strategies (IMTTS), Hannover Medical School, Carl-Neuberg-Str. 1, 30625 Hannover, Germany; Center for Translational Regenerative Medicine, Hannover Medical School, Carl-Neuberg-Str. 1, 30625 Hannover, Germany

**Keywords:** Sodium-glucose co-transporter 2 inhibitors, Cardiovascular diseases, Human living myocardial slices, Single-nucleus RNA sequencing, Heart failure

Over recent years, sodium-glucose co-transporter 2 inhibitors (SGLT2is) have emerged as effective treatments for patients suffering heart failure (HF), with dapagliflozin (DAPA) and empagliflozin (EMPA) headlining therapeutic recommendations in the current guidelines for HF management.^1,2^ Despite their unambiguous clinical benefits, it is still frequently refrained from usage of SGLT2is in many eligible patients^3^ presumably attributable to the incomplete understanding of the cardiovascular mode of action of these drugs. Multiple studies utilizing various *in vitro*, *ex vivo*, and *in vivo* models have been conducted to gather evidence on direct cardiovascular functions of SGLT2is. Those approaches have highlighted that SGLT2is are able to prevent adverse cardiac remodelling^4^ and improve contractile function through interference with ion transporters.^5^ However, precise knowledge of molecular events elicited by SGLT2is in the diseased human myocardium in its *in vivo* 3D composition remains lacking. To shed light on this, we herein utilized the organomimetic model of living myocardial slices^6^ in a miniaturized manner (mini-LMS) and performed single-nucleus RNA (snRNA) sequencing after therapeutic treatment with SGLT2is.

The mini-LMS were generated from myocardial tissue obtained during cardiac surgery of three different HF patients (*Figure 1A*). Sections of 4 mm length and width and a thickness of approximately 300 µm were prepared with a vibratome in cardioplegic buffer and stretched along muscle fibres using plastic scaffolds simulating mechanical load.^7^ Cultivation (at 37 °C, 5% CO_2_) under constant rocking to ensure optimal nutrient supply was performed for 24 h in the presence of either 100 µM DAPA or EMPA (both Selleckchem, Planegg, Germany) or respective dimethyl sulfoxide (Carl Roth, Karlsruhe, Germany) control. To obtain sufficient myocardial nuclei for subsequent RNA sequencing, two tissue pieces per group were combined and nuclei were isolated as described by Cui and Olson^8^ in steps four to eight applying 15 Dounce homogenizer strokes. Intact nuclei were stained with DAPI and extracted from the suspension with fluorescence-activated cell sorting (FACS) using a FACSAria (Becton Dickinson, Franklin Lakes, NJ, USA) based on fluorescence signal (450/20 filter) and side scatter. Library preparation for snRNA sequencing analysis was performed according to the Chromium NextGEM Single Cell 3ʹ Reagent Kits v3.1 User Guide (10× Genomics, Pleasanton, CA, USA). Libraries were quantified with the ‘Qubit® dsDNA HS Assay Kit’ (Q32854; Thermo Fisher Scientific) and diluted to 1.8 pM according to the ‘Denature and Dilute Libraries Guide’ (Illumina, San Diego, CA, USA). Sequencing was performed on an Illumina NextSeq550 sequencer using one high output flowcell kit for 75 cycles and 400 million clusters. Raw data were processed with the 10× Genomics CellRanger analysis pipeline set (v7.1.0) using default parameters. Subsequently, outputs from CellRanger count of all samples were aggregated, normalized to the same sequencing depth, and then the feature-barcode matrices were recomputed by CellRanger aggr. Seurat package for R (version 4.4.0) was used to analyse data. After removing cells with high mitochondrial gene content (>5%) or abnormal number of features (<200 or >2500), data were normalized and scaled. After dimension reduction, the cut-off for dimensionality was set at principal component 10.

**Figure 1:**
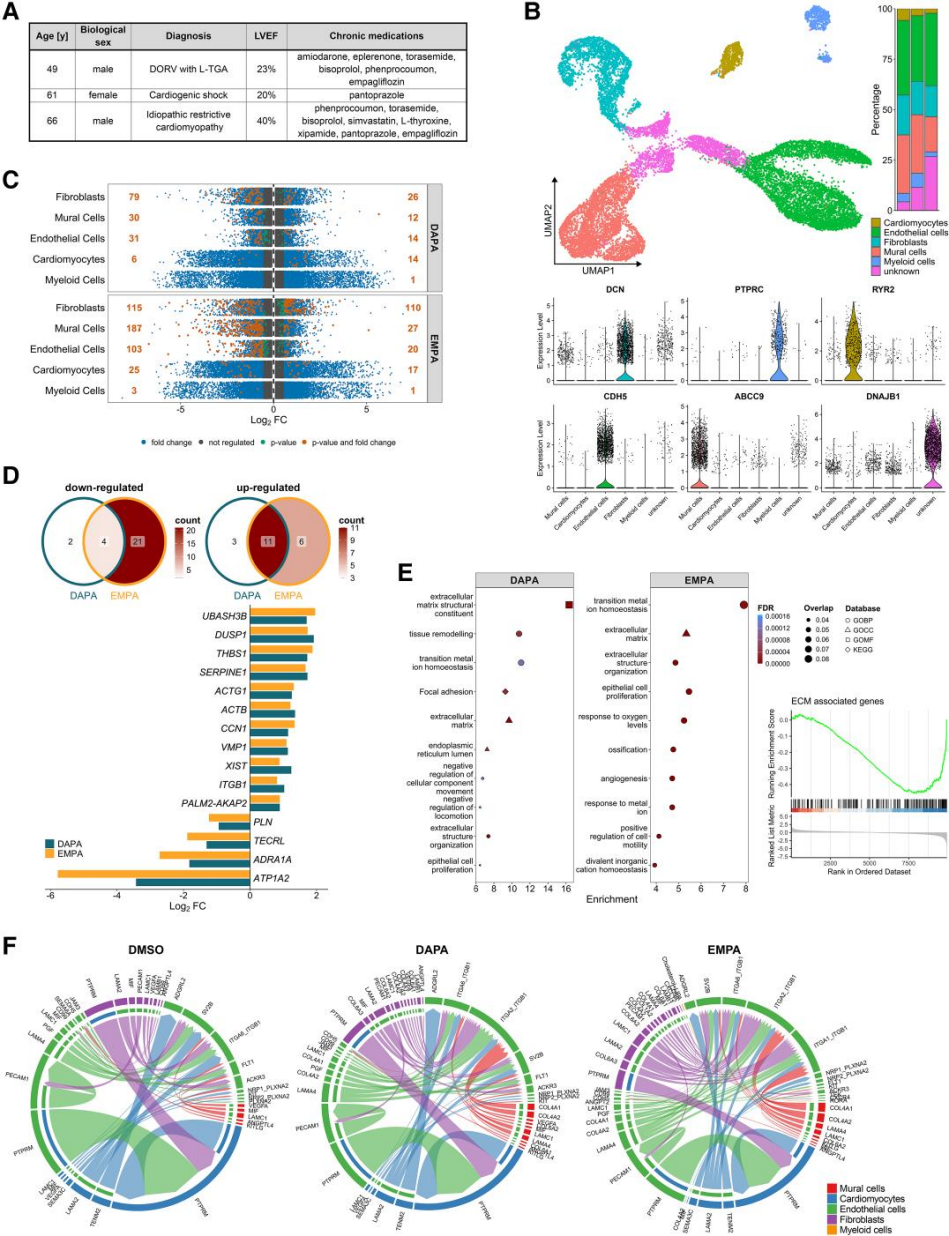
Cell-type–specific impacts of sodium-glucose co-transporter 2 inhibitors *ex vivo* underline cardiovascular benefits in heart failure patients. (*A*) Information of patients from whom tissue pieces were obtained for this study. (*B*) Uniform manifold approximation and projection plot of sequenced single nuclei from miniaturized living myocardial slices treated with dapagliflozin (DAPA), empagliflozin (EMPA), or respective dimethyl sulfoxide control (top left). Cell-type distribution for each of the three different donors is indicated on the right (top). Expression levels of representative marker genes of each identified cluster (bottom). The ‘unknown’ cluster showed high levels of cell stress-related genes and was excluded from further analyses. (*C*) Differentially expressed genes in each cell type in response to DAPA or EMPA treatment (averages from all donors). The cut-offs for average fold change (FC) and adjusted *P*-value were set at 1.5 and .05, respectively. (*D*) Venn diagrams overlapping differentially expressed genes in the cardiomyocyte fraction in response to DAPA and EMPA (top). Average FCs of parallel regulated genes (bottom). (*E*) Top 10 overrepresented terms in differentially expressed genes from outlined databases (left). Enrichment analysis of extracellular matrix-associated genes in the mural cell population of miniaturized living myocardial slices upon EMPA treatment (right). (*F*) Identified cellular communication networks in the sequencing dataset upon respective treatments using the CellChat database. BP, biological processes; CC, cellular component; DORV, double outlet right ventricle; ECM, extracellular matrix; FDR, false discovery rate; GO, gene ontology; KEGG, Kyoto Encyclopedia of Genes and Genomes; LVEF, left ventricular ejection fraction; MF, molecular function; TGA, transposition of the great arteries; UMAP, uniform manifold approximation and projection.

Cell-type clustering was performed with .1 resolution, and cell types were assigned to clusters based on canonical markers (*Figure 1B*). We isolated sufficient nuclei from viable mini-LMS for snRNA sequencing allowing identification of major cell types within the myocardial tissue. The largest portion comprised endothelial cells followed by mural cells, fibroblasts, and cardiomyocytes. One cluster termed ‘unknown’ exhibited high expression of cellular stress-associated genes, presumably induced by the tissue slicing procedure. Thus, this cluster was disregarded in further analyses. The distribution of remaining cell types was roughly comparable with freshly isolated tissue^9^ particularly when considering that donors presented end-stage HF.

Differential gene expression in response to DAPA or EMPA was calculated in scaled data with donor-specific effects being regressed out. All genes with fold change ≥ 1.5 and adjusted *P* ≤ .05 were deemed statistically significant. We observed most prominent effects in the three largest populations, i.e. endothelial cells, mural cells, and fibroblasts, while fewer genes were regulated in cardiomyocytes and myeloid cells (*Figure 1C*). Interestingly, EMPA showed stronger influence than DAPA in most cell types. Both SGLT2is strongly altered extracellular matrix (ECM)-associated gene expression in fibroblasts, such as down-regulation of several collagens, e.g. *COL1A2* and *COL3A1*, and *FN1* as well as *TIMP1* (*Figure 1E*). In line, the assessment of gene expression signatures in mural cells from EMPA-treated mini-LMS confirmed a down-regulation of expression of ECM genes (normalized enrichment score = −1.916, false discovery rate *q* = .0006) (*Figure 1E*). These results are in agreement with the anti-fibrotic effect of SGLT2is mentioned earlier^4^ and highlight cardiac tissue-specific effects on ECM production and remodelling in fibroblasts and mural cells as a putative underlying mechanism in human diseased heart muscle.

Following the preparation protocol, the morphological structure present in the myocardium *in vivo* is maintained in the herein developed mini-LMS model. This allows for meaningful assessment of intercellular communication patterns which we performed by applying the CellChat package for R. Comparing SGLT2i-treated mini-LMS with respective controls, we identified profound changes in signalling involving endothelial cells (*Figure 1F*). Of note, the SGLT2i-induced alterations in terms of ECM-involving processes were also reflected here suggesting an impact of SGLT2is on the vasculature through mural cells and fibroblasts.

Next, we investigated the effect of DAPA and EMPA on cardiomyocytes in more detail by filtering for genes that were regulated by both SGLT2is in parallel (*Figure 1D*). We found consistent down-regulation of genes interfering with Ca^2+^ signalling, a crucial effectory pathway mediating contractile function of cardiomyocytes. Selection included sarcoplasmic/endoplasmic reticulum calcium ATPase 2 inhibitor phospholamban (*PLN*) and sodium/potassium-transporting ATPase subunit α-2 (*ATP1A2*), which both have inhibitory effects on contractility. Regulation of dual specificity phosphatase 1 (*DUSP1*) and trans-2,3-enoyl-CoA reductase like (*TECRL*) suggested an influence of SGLT2is on mitochondrial homeostasis underlining the modulation of Ca^2+^ signalling and implying alterations in cardiomyocyte metabolism. In line with the latter, vacuole membrane protein 1 (*VMP1*), a strong inducer of autophagic processes, demonstrated two-fold up-regulation in response to SGLT2is recapitulating pro-autophagic observations made in HF patients receiving these drugs.^10^

Whether the herein reported effects of SGLT2is on the transcriptome of diseased human myocardium translate fully to the proteome and functional level remains to be clarified by future studies. The lack of functional readouts should be overcome in similar future studies by improving the methodology, e.g. via adding electrical stimuli and video-based options for contractility assessment. Moreover, inclusion of healthy control mini-LMS might aid to underline the clinical significance of the findings generated herein. Of importance, the transcriptomic fingerprints we observed in end-stage HF-derived human mini-LMS after SGLT2i application align with the clinically beneficial effects observed in treated patients and therefore can potently explain cardiovascular benefits of this drug class. We provide for the first time experimental evidence that viable ultrathin slices generated from diseased human heart tissue can be utilized to explore the effects of therapeutic interventions on a transcriptomic level with single-cell resolution in a highly translational way that can hardly be achieved by other models. Undoubtedly, this paves new avenues for pre-clinical drug research thereby enhancing rapid transition to the clinical setting. Taken together, we postulate the mini-LMS model system as a breakthrough for pharmacological, efficacy evaluations in cardiovascular drug development, and target identification approaches aiming to combat the detrimental burden of cardiovascular diseases.

## Data Availability

Single-nucleus RNA sequencing data can be obtained from the authors upon reasonable request.

## Pre-registered Clinical Trial Number

None supplied.
